# Analysis of neointima development in flow diverters using optical coherence tomography imaging

**DOI:** 10.1136/neurintsurg-2016-012969

**Published:** 2017-06-07

**Authors:** Yoshikazu Matsuda, Joonho Chung, Demetrius K Lopes

**Affiliations:** 1 Department of Neurological Surgery, Rush University Medical Center, Chicago, Illinois, USA; 2 Department of Neurosurgery, Wakayama Medical University, Wakayama City, Japan; 3 Department of Neurosurgery, Gangnam Severance Hospital, Yonsei University College of Medicine, Seoul, Republic of Korea

**Keywords:** Flow Diverter, Device, Intervention, Vessel Wall, Stent

## Abstract

**Background:**

Flow diverters are used for the treatment of intracranial aneurysms. Surface modification may decrease the thrombogenicity of flow diverters but the details are unknown. Optical coherence tomography (OCT) is an intravascular imaging test with high resolution which identifies neointimal growth over stents. We compared the development of neointima in a flow diverter and stents with and without surface modification in a swine model.

**Methods:**

In this study we implanted four devices (two in each carotid artery) in four pigs. The devices used were the Pipeline Flex embolization device (PED Flex, n=6), PED with Shield technology (PED Shield, n=6), and Solitaire AB (n=4). Serial carotid angiographic and OCT images were obtained on days 0, 7, 14, and 21. The data analyzed included: neointimal area (lumen area − stent area), neointimal ratio ([lumen area − stent area]/stent area), and the neointimal thickness ratio (minimum neointimal thickness/maximum neointimal thickness).

**Results:**

There was no significant difference in where neointima formation was initiated in relation to the implanted device (distal vs middle vs proximal). The PED Shield had a trend towards earlier endothelial formation at day 7. By day 21 the neointimal ratio was significantly higher for the PED Flex and PED Shield devices than for Solitaire (p<0.05 and p<0.01, respectively). The neointimal thickness ratio was significantly higher with PED Shield than with PED Flex and Solitaire (p<0.05 and p<0.01, respectively).

**Conclusions:**

OCT enabled us to follow and compare in vivo the development of neointima over implants. PED Shield showed a similar neointimal volume to PED Flex and more concentric neointima.

## Introduction

Flow diverters and the combination of stents and coils are commonly used for the repair of wide-necked intracranial aneurysms. Thromboembolic events are still one of the most common complications during endovascular interventions.^1–3^ Although the causes of thromboembolism are multifactorial, surface modification of the currently available Pipeline Flex embolization device (PED Flex; Medtronic, Irvine, California, USA) may reduce the thrombogenicity of this device. The new PED Flex device with surface modification is called PED with Shield technology (PED Shield) (Medtronic). The surface modification involves the incorporation of phosphorylcholine (PC) into the composition of the flow diverter. Previous experience with surface modification of coronary stents demonstrated no adverse effect on re-endothelialization with a decrease in the rate of stent thrombosis.^4–7^ Recently, one ex vivo study and one case report with single antiplatelet therapy using the PED Shield stent have been reported.^8^ ^9^

Optical coherence tomography (OCT) is an intravascular imaging technology with resolution capable of identifying neointimal growth over implanted intravascular devices. A number of reports have validated the correlation of OCT imaging and histology of the coronary arteries.^10^ ^11^

There have been no reports of re-endothelialization assessed by OCT imaging or histology with the PED Shield stent, although a previous study reported less thrombogenicity for this stent in vitro.^12^

The purpose of this study is to compare the difference in endothelialization between the PED Flex and Shield devices using OCT imaging under single antiplatelet therapy.

## Methods

### Animal study design

The study protocol was approved by the local institutional animal care and use committee. All animals received humane care in compliance with the Animal Welfare Act and the ‘Guide for the Care and Use of Laboratory Animals’ formulated by the Institute of Laboratory Animal Research (National Research Press, 1996).

### Procedural description

Six PED Flex devices, six PED Shield devices, and four Solitaire AB devices were implanted in the carotid arteries (two stents per vessel) of four Yorkshire pigs weighing 25–35 kg. The size of the implanted devices was 5×20 mm for PEDs and 4.0×20 mm for Solitaire AB. Three days before the endovascular procedure the animals were given 10 mg/kg of aspirin. Thereafter, aspirin was administered daily throughout the study. Our original protocol planned to use single antiplatelet therapy in all animals throughout the study. However, the first angiographic follow-up demonstrated a high rate of stent thrombosis so we decided to amend the protocol and start dual antiplatelet therapy. Clopidogrel 10 mg/kg was administered in all animals from day 8 after stent implantation. All thrombosed devices underwent successful recanalization after mechanical thrombectomy with aspiration and angioplasty with non-compliant balloons. Follow-up angiography showed no further implant thrombosis. During the study we performed intravascular images with OCT and angiography at four time points (days 0, 7, 14, and 21).

All animals were pre-anesthetized with a mixture of telazol and xylazine weight-based. After reaching an adequate anesthetic status, the animals were intubated and maintained with continuous inhalation of isoflurane (1–3%). A vascular access sheath (6 F) was percutaneously inserted into the femoral artery. After sheath insertion, heparin (3000–8000 U) was given through the sheath. Baseline angiography was acquired and two stents were implanted in each of the carotid arteries.

### OCT imaging protocol and analysis

**Figure 1:**
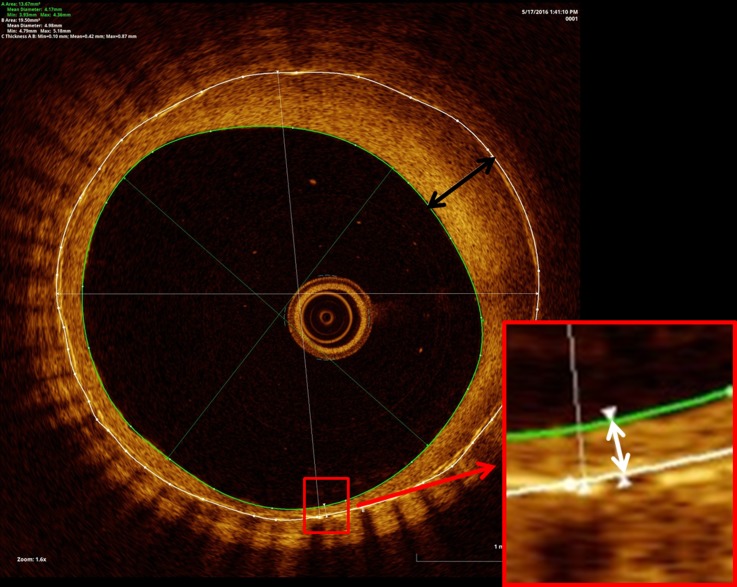
Automatic contour detection algorithm for carotid artery quantification. The endoluminal surface indicated by a green circle was automatically detected. Stent struts were manually traced and positioned in the center of the stent strut. The lumen, stent, and neointimal (stent − lumen) area were measured. Maximum and minimum thickness on magnification was automatically measured (black and white arrows).

OCT images were obtained using the Dragon fly Optis imaging system (LightLab Imaging, St Jude Medical, St Paul, Minnesota, USA). A motorized OCT catheter pull-back at a rate of 18.0 mm/s while a simultaneous IA iodine contrast injection at a rate of 4–6 mL/s for a total volume of 16–24 mL to clear the blood from the vessel were used to obtain all OCT images at 180 frames/s. Offline OCT image analysis was performed with Ilumien Optis post-processing software. We selected three cross-sectional images per pull-back (proximal stent, mid-stent, and distal stent) for each of the time points of the study. An automated contour detection algorithm was used for carotid artery quantification. The endoluminal surface was automatically detected. Stent struts were manually traced and positioned in the center of the stent strut which showed a bright ‘blooming’ appearance (figure 1).^13^ OCT images were excluded from the analysis if the stent struts and lumen contour were not visible.

The following parameters were measured (figure 1): lumen area (defined as contours of the vessel lumen), stent area (identified by circumferential area limited by the contours of the struts), neointimal area (stent area − lumen area), neointimal ratio ([mean stent area − mean lumen area]/mean stent area), and thickness ratio (minimum neointimal thickness/maximum neointimal thickness). The neointimal ratio could show the volume of neointimal formation and also restenosis. A higher ratio means more neointimal formation. The thickness ratio could reflect the concentricity of neointimal formation: it represents more concentric neointima formation when the ratio is close to 1 and eccentric neointima formation when the ratio is close to 0. To analyze neointimal thickness, the distance between the center of each stent and the luminal border was measured in the direction of the center of gravity. Maximum and minimum thicknesses were automatically calculated. Malapposition was defined as detachment from the vessel wall, considering stent strut thickness and blooming artifact of each type of stent. A total of 192 OCT images on days 0, 7, 14, and 21 were analyzed. Six OCT images were excluded because of poor images.

### Tissue harvesting and histology

Under anesthesia, all animals were euthanized immediately after the last follow-up imaging (day 21). The carotid arteries were excised and the stented carotid artery segments were harvested. After histological processing, the distal edge of the stent was marked. Three sections were chosen and correlated to OCT imaging at equidistance from the distal end of the stent. The specimens were post-fixed in 2.5% glutaraldehyde for a minimum of 2 hours. The slides created using the EXAKT system were stained with H&E.

A total of 48 OCT images on day 21 and histology-matched cross-sections were analyzed.

### Statistical analysis

Statistical analysis was performed using an add-in statistical analysis software Statcel V.3 (OMS Publishing Inc, Tokyo, Japan) for Microsoft Excel (Microsoft, Seattle, Washington, USA). The Student t-test, Fisher's exact test, two-way repeated measures analysis of variance (ANOVA), and one-way ANOVA were used for statistical comparisons. Tukey post hoc analysis was used. A p value of <0.05 was considered statistically significant.

## Results

**Figure 2:**
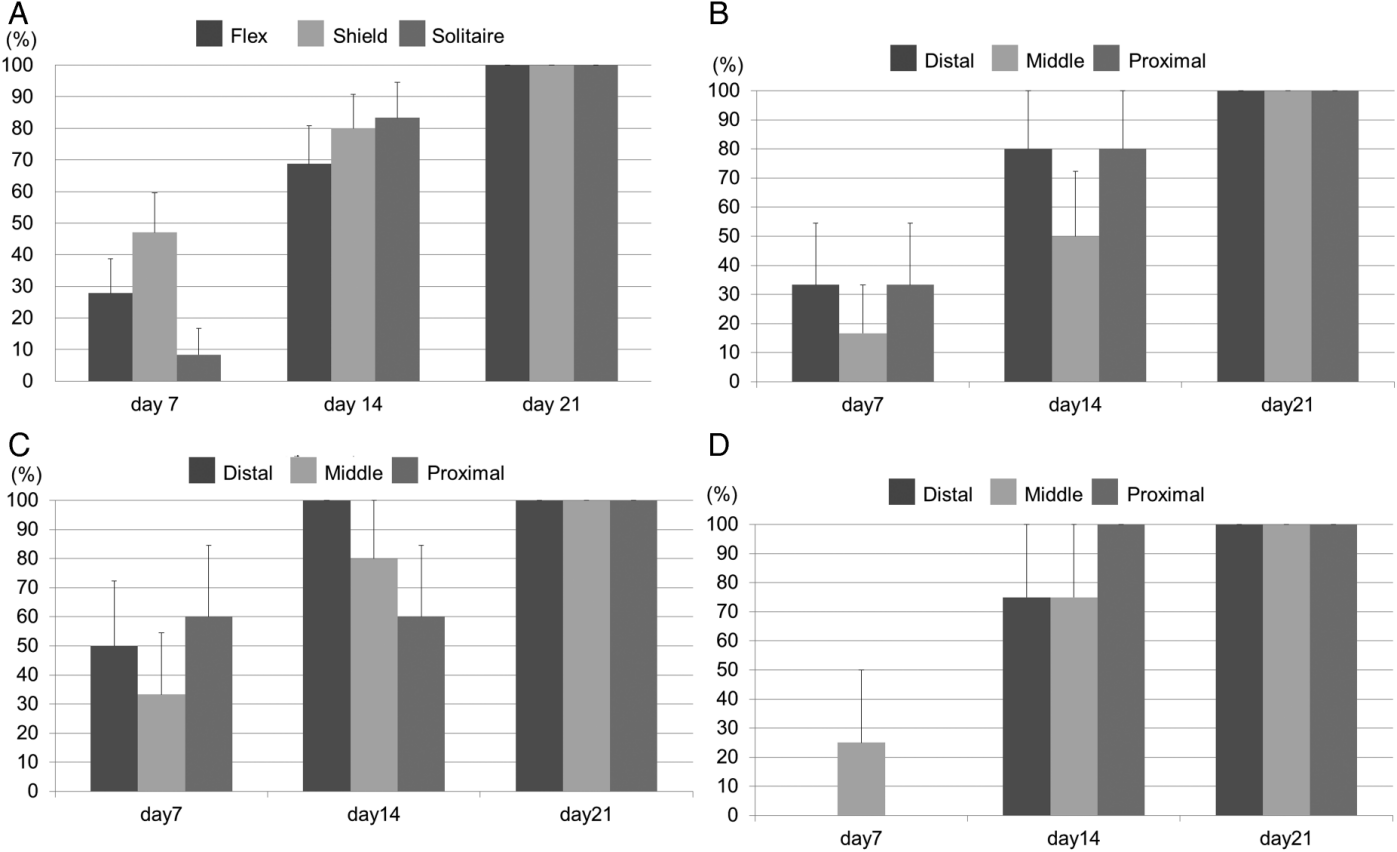
(A) Percentage neointimal formation by location over time did not differ significantly between stents. (B) Percentage neointimal formation of the Pipeline Flex embolization device (PED Flex) divided into distal, middle, and proximal portions. (C) Percentage neointimal formation of the PED Shield device in each position. (D) Percentage neointimal formation of the Solitaire device in each position. Each stent shows no difference between the three positions. Bars are +SEs.

Table 1 and figure 2A describe the neointima development for each stent during the study. The rate of neointima development for the PED Flex, PED Shield, and Solitaire devices on day 7 was 5/18 (27.8%), 8/17 (47.1%), and 1/12 (8.3%), respectively. On day 14, the rates were 11/16 (68.8%), 12/15 (80%), and 10/12 (80%), respectively. On day 21 all devices had complete neointima coverage.

**Table 1:** Results of OCT analysis in each stent

	PED Flex				PED Shield				Solitaire			
Implanted stents, n	6				6				4			
Thrombosed stents on day 7, n	6				2				0			
Position-based analysis	n=18				n=18				n=12			
	**Day 0**	**Day 7**	**Day 14**	**Day 21**	**Day 0**	**Day 7**	**Day 14**	**Day 21**	**Day 0**	**Day 7**	**Day 14**	**Day 21**
Lumen area, mm^2^*	16.53±2.12	9.67±3.50	11.12±4.12	10.09±3.87	15.68±1.02	10.01±2.73	9.15±3.98	8.99±4.01	12.68±4.78	8.47±3.88	8.69±2.38	8.82±2.53
Mean diameter, mm*	4.57±0.31	3.42±0.65	3.69±0.72	3.52±0.69	4.47±0.15	3.52±0.52	3.33±0.68	3.31±0.76	3.94±0.78	3.21±0.71	3.30±0.43	3.07±0.93
Stent area, mm^2^	15.95±1.99	13.51±3.55	13.38±4.1	12.13±4.54	12.12±2.80	12.12±2.80	11.42±4.86	11.71±5.58	8.86±3.74	8.87±3.74	9.23±1.88	9.30±1.79
Mean diameter, mm	4.50±0.29	3.89±0.92	4.08±0.63	3.86±0.74	4.38±0.18	3.90±0.43	3.74±0.76	3.76±0.91	3.79±0.65	3.29±0.67	3.41±0.35	3.42±0.34
Neointima, mm^2^ (stent − lumen)		3.84±3.02	2.26±1.37	2.04±1.73		2.11±3.36	2.26±2.23	2.72±2.06		0.40±0.39	0.54±0.93	0.48±1.22
Neointimal ratio ([stent − lumen]/stent)				0.16±0.11				0.21±0.096				0.064±0.12
Neointimal thickness minimum, mm				0.039±0.04				0.077±0.071				0.0042±0.012
Neointimal thickness maximum, mm				0.39±0.31				0.45±0.33				0.26±0.16
Thickness ratio (min/max)				0.1±0.12				0.21±0.17				0.022±0.068
Detected neointima in every position, %		27.8	68.8	100.0		47.1	80.0	100.0		8.3	83.3	100.0
Distal position, %		33.3	80.0	100.0		50.0	100.0	100.0		0.0	75.0	100.0
Middle position, %		16.7	50.0	100.0		33.3	80.0	100.0		25.0	75.0	100.0
Proximal position, %		33.3	80.0	100.0		60.0	60.0	100.0		0.0	100.0	100.0

Data are reported as mean±SD.

*p<0.05 PED Flex vs Solitaire stent.

OCT, optical coherence tomography; PED, Pipeline Flex embolization device.

OCT was used to monitor whether, over time, neointima developed from the edges (proximal or distal) or from the centre (middle) of the implanted devices. We found that neointima coverage of the devices did not follow a predictable pattern of development (table 1, figure 2B–D).

**Figure 3:**
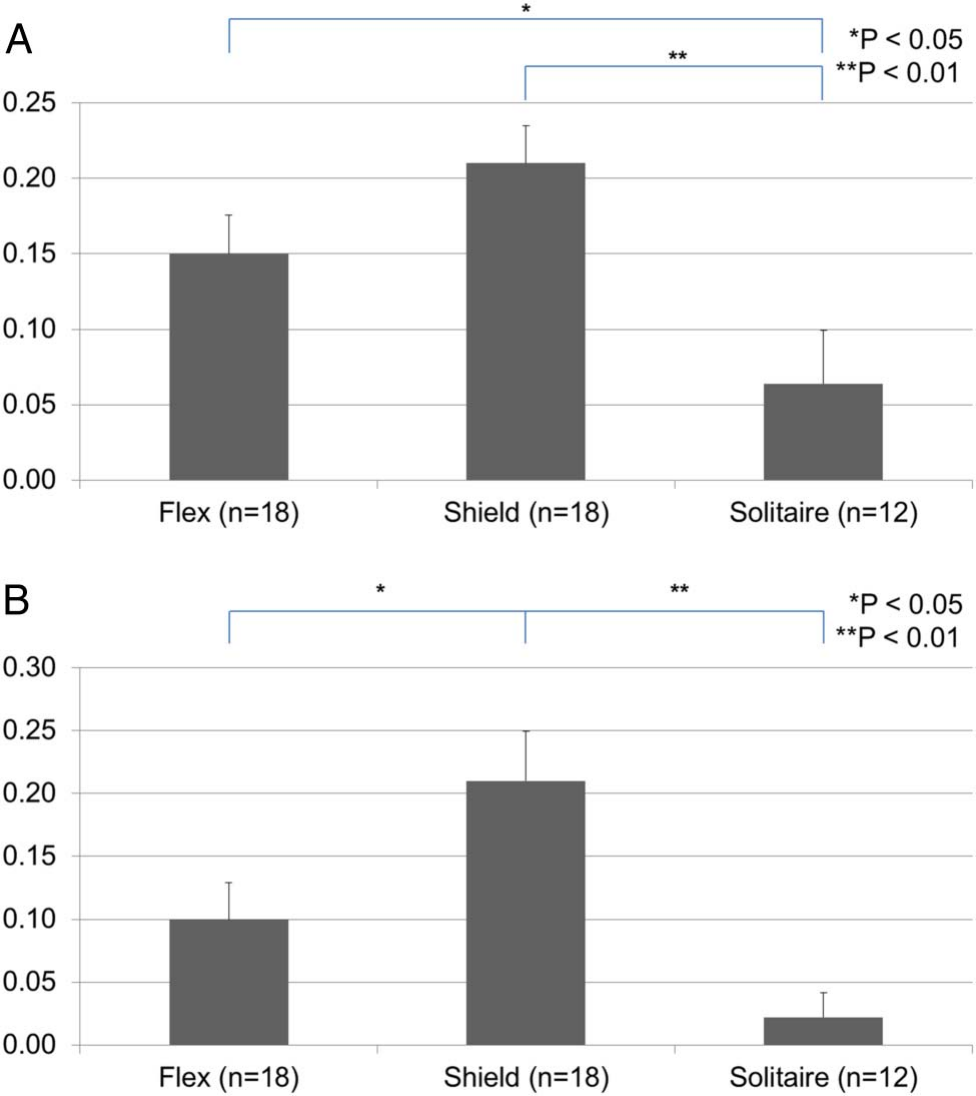
(A) Neointimal ratio on day 21 in each stent. The Pipeline Flex embolization device (PED Flex) and PED Shield stent had a significantly higher neointimal ratio than the Solitaire stent (p<0.01 and p<0.05, respectively). There is no difference between the PED Shield and PED Flex. (B) Neointimal thickness ratio on day 21 in each stent. The PED Shield stent shows a significantly higher neointimal thickness ratio than the PED Flex and Solitaire stents (p<0.05 and p<0.01, respectively). There is no difference between the PED Flex and Solitaire stents. Bars are +SEs.

The neointimal ratio was significantly higher in the PED Flex and PED Shield groups than in the Solitaire group on day 21 (p<0.05 and p<0.01, respectively). There was no difference between the PED Flex and PED Shield groups (figure 3A). The neointimal thickness ratio on day 21 was significantly higher in the PED Shield group than in the PED Flex and Solitaire groups (p<0.05, p<0.01; figure 3B).

**Figure 4:**
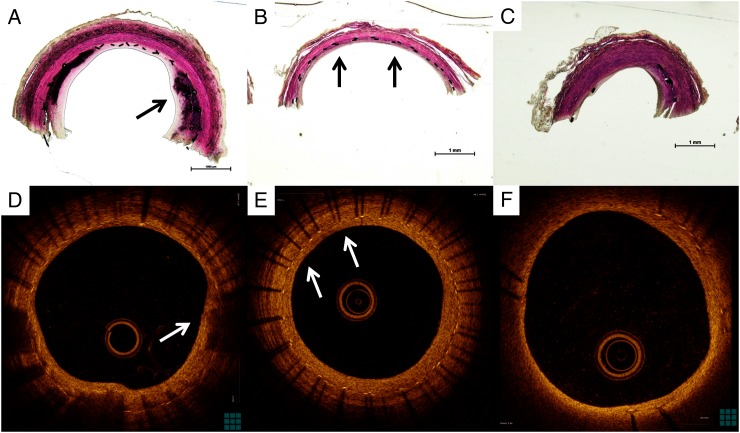
Representative optical coherence tomography (OCT) images and upper part of the histopathological findings divided longitudinally in each stent. (A) Asymmetrical neointima in the Pipeline Flex embolization device (PED Flex). Black arrow shows neointima covering mineralization. (B) Symmetrical neointima in the PED Shield stent (black arrows). (C) Asymmetrical neointima and less stent strut in Solitaire stent. (D) OCT image of asymmetrical and heterogeneous neointima in the PED Flex stent. White arrow shows neointima covering low back scattering lesion. (E) OCT image of symmetrical and homogenous neointima in the PED Shield stent (white arrows). (F) OCT image of asymmetrical and homogeneous neointima in the Solitaire stent.

OCT images correlated precisely with histopathological preparations, as shown in figure 4. PED Flex demonstrated asymmetrical neointima (figure 4A, D). The histopathological findings correlation shows neointima covered above mineralization. The Solitaire stent also had an asymmetrical neointima development (figure 4C, F) while the PED Shield had more symmetrical neointima growth (figure 4B, E).

The mean lumen diameter of the carotid artery on angiography before deployment of the PED Flex, PED Shield, and Solitaire stents was 4.58±0.39 mm, 4.40±0.22 mm, and 4.61±0.32 mm, respectively. The mean lumen diameter of the carotid artery in these three groups on OCT imaging after deployment of the stent was 4.57±0.31 mm, 4.47±0.15 mm, and 3.94±0.78 mm, respectively. The lumen diameter in the Solitaire group showed a significant reduction after deployment of the stent (p=0.012). A possible reason is that spasm may have occurred when the Solitaire stent was deployed.

During the acute phase after device implantation in pigs taking single antiplatelet therapy (aspirin) there were 8/16 thrombosed devices (6/6 PED Flex, 2/6 PED Shield). The PED Flex group had significantly more thrombosed devices than the PED Shield and Solitaire stent groups (p<0.05 and p<0.05, respectively). No further thrombosed devices were seen after instituting dual antiplatelet therapy.

## Discussion

This study illustrates a novel method of comparing the tissue response (healing) of intravascular implants. The use of OCT intravascular imaging allowed us to follow and compare in vivo the development of neointima over implants with different characteristics. The impact on device ‘healing’ of surface modification of flow diverters with PC polymer is unknown.

A previous study reported no adverse effect on the rate of re-endothelialization in coronary stents, although theoretically the PC-coated stent might induce a reduction in the rate of re-endothelialization because of inhibition of deposition of plasma protein onto the stent strut.^5^ ^14^ Our result was similar to this report. The percentage of neointimal formation each week in the PED Shield group was not significantly different from the PED Flex group (figure 2A), although the PED Shield group showed a tendency towards a higher percentage of neointimal formation than the PED Flex group. Although the mechanism for the effect of PC coating on wound healing and neointimal formation was unknown in previous reports,^5^ we hypothesize that PC coating on the PED Shield device might result in an earlier healing response because PC could shorten a few early steps of neointima formation. On the other hand, the neointimal ratio in the PED Flex and PED Shield groups was significantly higher than in the Solitaire group. We suggest that this difference in the effect on neointimal formation was due to the fact that there are more stent struts in flow diverters than in the Solitaire device, resulting in more ‘support’ for the development of the neointima. Darsaut *et al* reported that aneurysm occlusion was associated with neointimal closure of the flow diverter pores and the amount of metal surface coverage over the aneurysm ostium.^15^ Our study shows that flow diverters (PED Flex and PED Shield) have earlier as well as thicker neointimal formation than flexible stents (Solitaire). This result illustrates why flow diverters induce more durable aneurysmal occlusion than intracranial flexible stents. Surface modification with PC may be an independent factor on neointima development since we observed that the thickness ratio in the PED Shield group was significantly higher than in the PED Flex and Solitaire groups. Shibuya *et al*^16^ reported that eccentric and heterogeneous neointima formation in coronary stents assessed by OCT could signal late neointimal hyperplasia. The OCT images in the PED Flex group showed an eccentric and heterogeneous growth pattern while the PED Shield had a concentric and homogeneous neointimal pattern of growth. In our study the PED Flex was associated with more thrombus formation on the struts. The histology revealed that the neointima developed over the thrombus on the struts, perhaps resulting in the eccentric and heterogeneous pattern seen in the PED Flex group. Figure 4A, D shows the thrombus on the stent struts covered by neointima (also described as mineralization). The effect of PC surface modification might result in less thrombus and a more concentric neointima formation resulting in less late neointimal hyperplasia and reducing in-stent stenosis rates.

A few histological reports have shown that the initiation of neointimal formation for implanted coronary bare metal stents in pigs is 7 or 14 days.^17^^–^20 Many studies also showed a correlation between OCT imaging and histological evaluation.^10^ ^11^ Virmani *et al* reported surface endothelialization of >70% at 7 days. In this study the initiation of neointimal formation on the devices studied using OCT imaging was demonstrated by day 7 (figure 2). However, the endothelialization rate was different between the Solitaire stent and bare metal stents in a previous report. The difference between OCT imaging and histopathology might be one possible reason. OCT imaging may not be detectable if neointima on the surface of the stent is very thin, especially <10 µm.

We divided the implants into three sections (distal, middle and proximal) and evaluated where neointima formation was initiated in each section (figure 2). No significant difference was found between the three positions. de Prado *et al*^17^ and Virmani *et al*^18^ reported that the inflammatory reaction containing fibrin and platelets or macrophages around stent struts were factors for initiation of neointimal formation. Furthermore, de Prado *et al*^17^ reported that no significant differences were detected although the endothelialization of the stent struts at the edges (both proximal and distal) seemed to be better than in the middle part of the coronary stent. We observed a tendency for better neointima formation in the PED Shield and PED Flex groups at both the distal and proximal ends of the stent compared with the middle of the stent, but this finding was not statistically significant (figure 2B, C). We concluded that neointimal formation starts at random sites within the implanted device.

This study had several limitations. We performed mechanical thrombectomy for thrombosed devices. Any intravascular manipulation before or after device implantation can damage the initial endothelial coverage. As a result, this manipulation might lead to misinterpretation of the extent of final tissue coverage. Although a recent animal study suggests that mechanical thrombectomy with aspiration alone did not cause endothelial damage,^21^ angioplasty can result in full de-endothelialization of the vessels.^22^ ^23^ Another limitation is that six PED Flex and two PED Shield vessels thrombosed at day 7, resulting in an even further effect from interaction of the clot with the vessel wall. This biological effect can alter the process of neointimal formation. The results of our study should be interpreted in light of the limitations of our porcine model. All the treated vessels were straight and without aneurysms or branches. This model does not evaluate some of the hemodynamic forces and challenges of aneurysm neck healing seen in clinical situations.

## Conclusion

This study illustrates a novel method of comparing the tissue response (healing) of intravascular implants. The use of OCT intravascular imaging allowed us to follow and compare in vivo the development of neointima over implants with different characteristics. The PED Shield device showed a tendency for earlier neointimal formation. The volume of neointimal formation for the PED Shield stent on day 21 was not significantly different from that for the PED Flex. However, the PED Shield showed more evenly distributed concentric neointimal formation than the other devices. The pattern of neointima formation over an implant is random.
